# Transcriptome Response to Drought, Rehydration and Re-Dehydration in Potato

**DOI:** 10.3390/ijms21010159

**Published:** 2019-12-25

**Authors:** Yongkun Chen, Canhui Li, Jing Yi, Yu Yang, Chunxia Lei, Ming Gong

**Affiliations:** 1 School of Life Sciences, Yunnan Normal University, Kunming 650550, China; 2 Joint Academy of Potato Science, Yunnan Normal University, Kunming 650550, China

**Keywords:** potato, drought, rehydration, drought hardening, memory

## Abstract

Potato is an important food crop and its production is susceptible to drought. Drought stress in crop growth is usually multiple- or long-term. In this study, the drought tolerant potato landrace Jancko Sisu Yari was treated with drought stress, rehydration and re-dehydration, and RNA-seq was applied to analyze the characteristics of gene regulation during these treatments. The results showed that drought-responsive genes mainly involved photosynthesis, signal transduction, lipid metabolism, sugar metabolism, wax synthesis, cell wall regulation, osmotic adjustment. Potato also can be recovered well in the re-emergence of water through gene regulation. The recovery of rehydration mainly related to patatin, lipid metabolism, sugar metabolism, flavonoids metabolism and detoxification besides the reverse expression of the most of drought-responsive genes. The previous drought stress can produce a positive responsive ability to the subsequent drought by drought hardening. Drought hardening was not only reflected in the drought-responsive genes related to the modified structure and cell components, but also in the hardening of gene expression or the “memory” of drought-responsive genes. Abundant genes involved photosynthesis, signal transduction, sugar metabolism, protease and protease inhibitors, flavonoids metabolism, transporters and transcription factors were subject to drought hardening or memorized drought in potato.

## 1. Introduction

Potato is the most important food crop after wheat and rice [1]. It is more sensitive to water deficit than other crops, and if it suffers from drought during the critical growth period, its yield and quality will decrease significantly [2,3]. Due to climate change and the expansion of potato cultivation area in drought prone areas, the impacts of drought on potato production will continuously increase [4,5]. Improving the drought resistance of potato and breeding drought-resistant varieties are important strategies to reduce these negative impacts. Profiling of drought-resistance mechanisms and identification of drought-resistance genes are helpful ways to achieve this goal.

Plant drought-resistance is regulated by complex gene networks. Analysis of leaf cDNA gene chip showed that drought stress increased mitochondrial metabolic activity and inhibited photosynthesis-related genes in drought-tolerant potato [6,7]. Transcriptome analysis showed that transcription factors, protein kinases, proteins related to redox regulation, carbohydrate metabolism and osmotic adjustment participate in the tolerance of potato to osmotic stress [8,9,10]. Twenty-three drought-responsive genes were identified by comparing the transcriptomes of drought-tolerant and drought-sensitive potato varieties under water stress, and seven homologous genes were identified by homozygous mutants of *Arabidopsis thaliana*, six of them including carbohydrate transporter, mitogen-activated protein kinase kinase kinase 15 (*MAPKKK15*), serine-like carboxypeptidase 19 (*SCPL19*), armadillo/beta-catenin-like repeat-containing protein, high-affinity nitrate transporter 2.7 and nonspecific lipid transfer protein 2 (*nsLPT*) can improve the drought resistance of *Arabidopsis* [11]. These results suggest the complexity of the molecular mechanism of potato drought-resistance. Moreover, drought stress will have positive impacts on the subsequent stress, and drought hardening can improve the drought resistance of plants [12,13]. The adaptation of plants to repeated and long-term drought showed up stress memory [4]. Some genes will have memory effect on stress in this process and will show higher expression level when drought occurs again. For example, response to dehydration 29B (*RD29B*) and response to ABA 18 (*RAB18*) in *Arabidopsis* [14], 120 genes in coffee including resistant protein RGA2, gibberellin dioxygenase 1 and gibberellin oxidase 1 [15], and also lncRNA, DNA methylation and endogenous phytohormones (especially abscisic acid) participate in rice short-term drought memory [13]. Potato can also adapt to drought through repeated and long-term drought [16,17].

Diploid potato species have a wide genetic background, making it easier to isolate drought-resistant genes and analyze the molecular mechanisms of drought resistance, which can be as an effective material for drought resistance research in potato [10]. Jancko Sisu Yari (JSY) is an excellent drought-resistant germplasm of diploid Andean landraces. After drought stress, its yield only decreased by 6.3% compared with that of the control group [18]. It also has good frost resistance and certain antivirus, antinematode and antihail ability [19]. In this study, transcriptome analysis of JSY under continuous treatments of dehydration/rehydration/re-dehydration was performed for profiling the response to drought, rehydration, and re-dehydration stress and for elaborating the molecular mechanism of drought resistance in potato more comprehensively.

## 2. Results

### 2.1. Overview of Transcriptome Sequencing and Mapping to the Reference Genome

There were six groups of treatments, the first mild drought stress (D1), first severe drought stress (D2), re-watered (RW), mild re-dehydration (RD1), severe re-dehydration (RD2) and control (C0); each group included three replicates and a total of 18 samples were sequenced. Approximately 1.39 Gbp clean reads and 208.6 Gbp were generated, with an average of 11.6 Gbp per sample. The high-quality (HQ) clean reads account for 99.03%; Q30 (0.1% error rate) was 95.96% and the average GC content was 43.86%. The ribosomal RNA removed reads were mapped against the reference genome *S. tuberosum* Group Phureja DM1-3 PGSC v4.03 with a mapping rate of 63.65% and 59.78–62.46% unique mapping reads proportion (Table S1).

### 2.2. Differential Expression Analysis of Genes Responding to Dehydration, Rehydration and Re-Dehydration

Differential expression analysis was based on fragments per kilobase of transcript per million fragments mapped reads (FPKM). The threshold of differentially expressed gene (DEG) was set at log2 fold change of FPKM (log2FC) ≥ 1 and false discovery rate (FDR) ≤0.05. The DEG numbers are shown in Table 1. Here, 1329 DEGs were detected in first mild drought treatment (D1). With the increase of water loss, the amount of DEGs between C0 and D2 was increased to 3203. Compared with RW, there were 461 DEGs in RD1 and 1772 DEGs in RD2. Compared with the control, there were still 308 DEGs in rehydration (Table 1).

**Figure 1:**
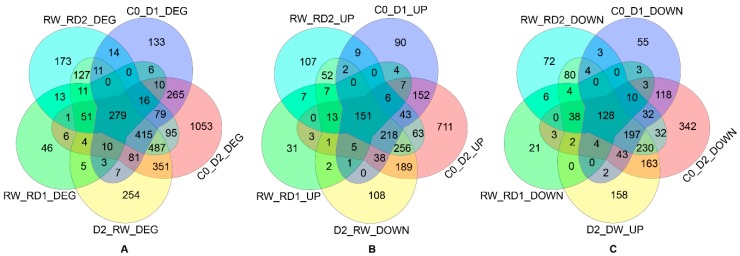
(**A**) differentially expressed genes (DEGs) in drought, rehydration and re-drought treatments; (**B**) DEGs of upregulated in drought and re-drought treatment and downregulated in rehydration; (**C**) DEGs of downregulated in drought and re-drought treatment and upregulated in rehydration.

Most (87.88%) of the DEGs compared between RW and D2 were differential expressed in response to drought (C0 vs. D1, C0 vs. D2, RW vs. RD1 and RW vs. RD2). Among them, 83.51% (172) of the downregulated genes after re-water (RW) were recuperative DEGs of D2, while the upregulation of 76.45% (248) upregulated genes after re-water was the recovery of downregulated DEGs compared between C0 and D2 (Figure 1). It is implied that most of the genes responding to drought stress can be recovered, that is, after drought stress, most of the up- or downregulated genes showed opposite regulation status after rehydration.

### 2.3. GO Enrichment of DEG

**Figure 2:**
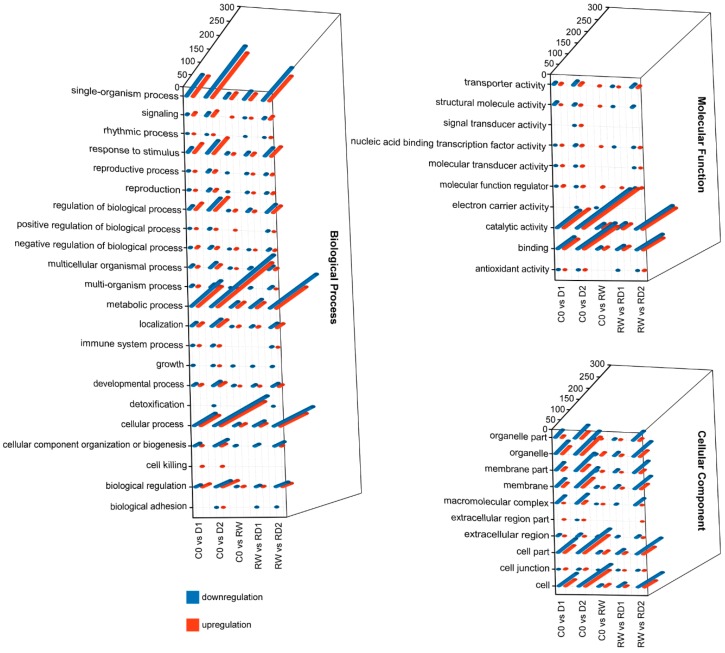
The gene ontology (GO) enrichment of DEGs in response to drought and re-dehydration in potato. The blue columns indicate the numbers of downregulation genes, while the red columns indicate the numbers of upregulation genes.

Gene ontology (GO) enrichment analysis showed that the number of DEGs increased with the increase of drought intensity, the number of DEGs between rehydration and control was less than that of D1 or D2 compared to C0 and RD1 or RD2 compared to RW (Figure 2). In the classification of biological process (GO:0008150), the numbers of DEGs enriched in single-organism process (GO:0044699), metabolic process (GO:0008152), cellular process (GO:0009987), biological regulation (GO:0065007) and response to stimulus (GO:0050896) were significantly greater than the other classifications. In the molecular function (GO:0003874) classification, more genes were enriched in the category of catalytic activity (GO:0003824) and binding (GO:0005488). In the cellular component (GO:0005575) classification, extracellular region (GO:0005576) and extracellular region part (GO:0044421) were classifications with the least genes. Among most of categories, the number of downregulated genes is greater than the number of upregulated genes. However, in signaling (GO:0023052) and response to stimulus (GO:0050896) the number of upregulated genes is greater than the number of downregulated genes in drought response. This suggests that these two classes of genes may play important roles in drought resistance.

The regulation of biological process (GO:0050789), biological regulation (GO:0065007) and catalytic activity (GO:0003824) showed that upregulated genes were more prevalent than downregulated genes in the first drought, while the opposite results were seen after re-hydration. The entries showed significant differences in gene expression between the two drought processes.

### 2.4. KEGG Enrichment of DEGs

**Figure 3:**
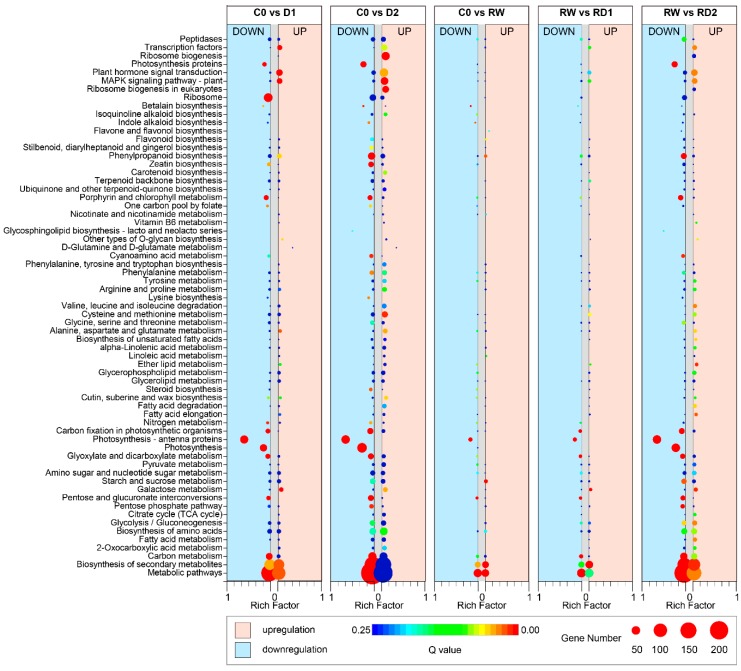
Kyoto Encyclopedia of Genes and Genomes (KEGG) enrichment of DEGs in response to drought and re-dehydration in potato. The area of bubbles indicates the number of enriched DEGs, while the color of bubbles indicates Q-value. Those on the light pink background are the enriched upregulated DEGs; the downregulated DEGs are on the light blue background.

KEGG (Kyoto Encyclopedia of Genes and Genomes) enrichment showed that DEGs in drought stress response involve a large number of pathways (Figure 3). With the aggravation of drought stress, the number of enriched DEGs increased; and there were more DEGs in the first drought than in re-dehydration. Similar to GO enrichment, the enriched DEGs between rehydration and control was less than that in response to drought and re-dehydration. The highest enrichment factors among the comparison pairs of C0–D1, C0-D2, C0–RW, RW–RD1 and RW–RD2 all were photosynthesis-related pathways such as photosynthesis-antenna proteins, photosynthesis, photosynthesis proteins and porphyrin and chlorophyll metabolism. Most of these DEGs were downregulated under drought and re-drought stress. The response of galactose metabolism, alanine/aspartate/glutamate metabolism, plant hormone signal transduction, plant MAPK signaling pathway and transcription factors (TFs) pathways were significantly upregulated in the first drought, and with the increase of water loss, the number of upregulated genes increased. Though the responsiveness degree decreased significantly when the drought occurred again, the responses were still obviously induced by drought. Cysteine and methionine metabolism, fatty acid metabolism, cutin/suberine/wax biosynthesis and ribosome biogenesis pathways also showed some similarities. These indicated that most of the DEGs in these pathways were continuously induced by drought. The responses of starch and sucrose metabolism and phenylpropanoid biosynthesis pathways were significant during rehydration, indicating that DEGs play important roles in rehydration. These two pathways were induced by light drought, but were suppressed with the aggravation of drought, and the upregulated DEGs were more prevalent than downregulated ones in mild drought, with the opposite occurring in severe drought.

### 2.5. Drought-Responsive Genes

**Figure 4:**
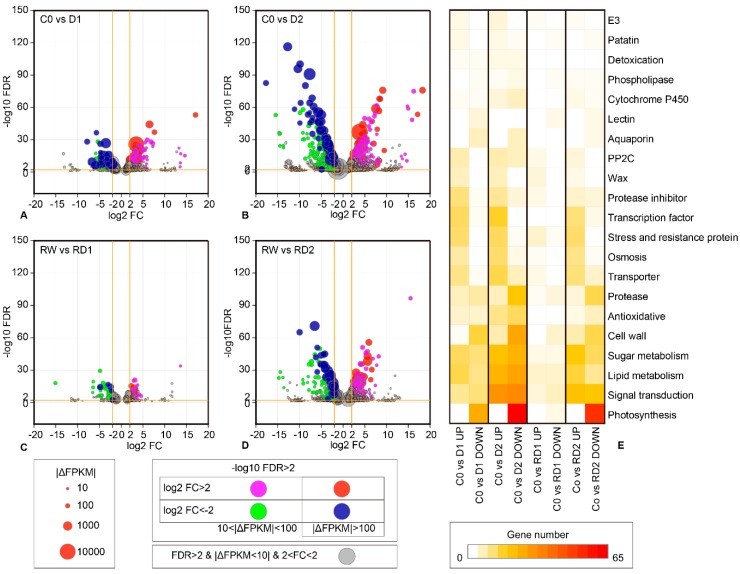
Drought-responsive DEGs between (**A**) C0 and D1, (**B**) C0 and D2, (**C**) RW and RD1, (**D**) RW and RD1. (**E**) The DEG numbers heatmap of primary drought-related genes. The area of bubbles indicates the expression level difference of genes between the compared pair (|ΔFPKM|). The grey bubbles indicate the genes with low expression (|ΔFPKM| < 10) or small and insignificant differences (−2 < log2FC < 2 or −log10FDR < 2), while colorful bubbles indicate genes with greater expression level and fold change (|ΔFPKM| > 10; |log2FC| > 2; −log10FDR > 2), among them, the red and blue bubbles indicate |ΔFPKM| > 100.

Compared with the control, the number of DEGs in D2 was greater than that in D1; the number DEGs of RD2 was also more than that of RD1, compared to re-water (Figure 1), which showed that the number of DEGs increases with the increase of drought. With the increase of drought, not only was the number of DEGs with high expression abundance (|ΔFPKM| > 10) increased, but also the fold change was enlarged (Figure 4). Plenty of DEGs related to photosynthesis, signal transduction, lipid metabolism, glucose metabolism and other functions were involved in drought and re-drought response (Table S2). Among them, the DEGs related to photosynthesis were obviously inhibited, and the number of downregulated genes of RD1 was obviously less than D1; there were up to 80 downregulated, such as photosystem I (PSI), photosystem II (PSII), chlorophyll a–b binding protein gene (*CAB*), protochlorophyllide reductase, and so on (Table S2). Protein phosphatase 2C (*PP2C*), waxy-synthesis-related genes, transcription factors, resistance-related genes, osmotic adjustment genes and transporter genes were mostly induced by drought. There were more downregulated protease genes than upregulated, while the opposite was the case for protease inhibitors (Table S2, Figure 4E).

Among those drought-response genes, some classes of genes showed obvious convergence. For example, most of the nonspecific lipid transfer proteins (*nsLTP*) were upregulated. Most of the upregulated drought-responsive expansin genes were expansin-like genes (*EXLA* and *EXLB*). Abundant genes involved in osmotic adjustment were upregulated, such as osmotin-like (*OSML*), delta-1-pyrroline-5-carboxylate synthase (*P5CS*), late embryogenesis abundant protein (*LEA*) and galactinol-sucrose galactosyltransferase (raffinose synthase, *RFS*). Most of the transcription factors were induced by drought, and the upregulated genes mainly included most of *MYB*, *WRKY*, *NAC* and *ERF*. The downregulated TFs were mainly concentrated in *bHLH* and CONSTANS-like zinc finger proteins. The upregulated hormone signal transduction related genes were mainly involved in abscisic acid (ABA) and ethylene signal transduction genes such as serine/threonine-protein kinase *SAPK2*, protein phosphatase 2C (*PP2C*), 1-aminocyclopropane-1-carboxylate oxidase (*ACO*) and ethylene-responsive transcription factor (*ERF*), while the genes related to auxin, zeatin and gibberellin such as auxin-binding protein (*ABP*), auxin-responsive protein, zeatin O-glucosyltransferase (*ZOG*), zeatin O-xylosyltransferase (*ZOX*), gibberellin 20 oxidase (*GA20OX*) and GA-stimulated transcript 1 (*GAST1*) were downregulated.

### 2.6. DEGs after Rehydration

One day after rehydration, 1053 genes were upregulated (log2FC > 1) compared with D2, of which 806 (76.54%) were downregulated in C0–D2, and 1044 genes were downregulated, of which 872 (83.52%) were upregulated in C0–D2, suggesting that the expression of most genes in response to drought stress tended to recover to the control after rehydration.

**Figure 5:**
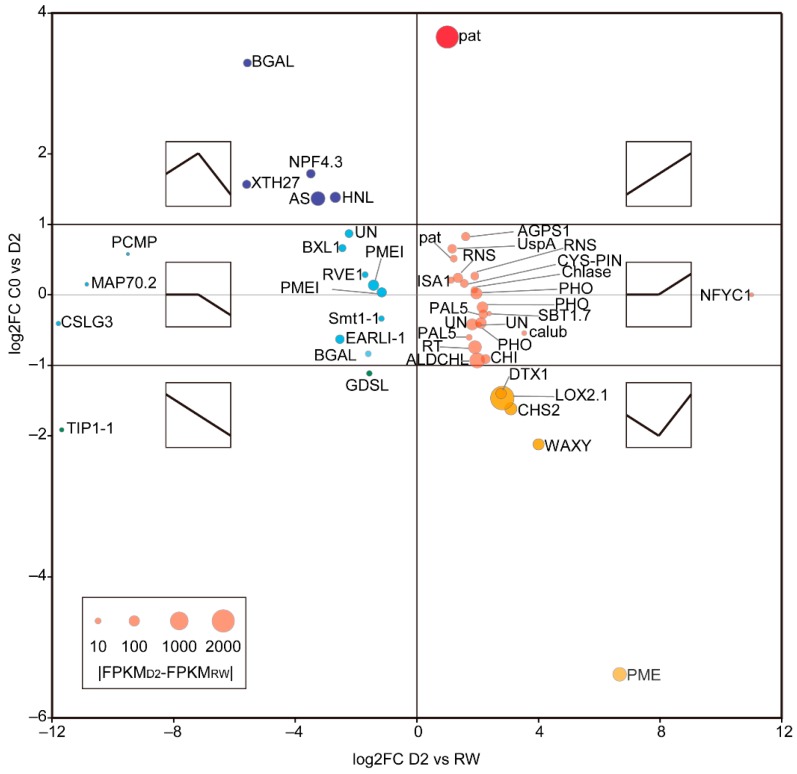
DEGs after rehydration compared with C0 and D2. The red and orange bubbles indicate upregulated genes with C0 and D2, while the blue and green indicate downregulated genes. The area of the circle indicates the difference of expression between D2 and RW. The line charts in the small square frame are expression trends of genes in the coordinate region.

After re-water, 27 genes were upregulated and 18 genes were downregulated compared to C0 and D2 (Figure 5, Table S3). These 27 upregulated genes included patatin (pat), linoleate 13S-lipoxygenase (*LOX2.1*), fructose-bisphosphate aldolase (*ALDCHL*), phenylalanine ammonia-lyase (*PAL5*), alpha-glucan phosphorylase (*PHO*), pectinesterase-like (*PME*), anthocyanidin 3-O-glucosyltransferase (*RT*), protein detoxification (*DTX1*), chalcone synthase (*CHS2*) and granule-bound starch synthase (*WAXY*); these genes were mainly involved in fat acid metabolism, sugar metabolism, flavonoid metabolism and detoxification. The downregulated genes included asparagine synthetase (*AS*), hornerin-like (*HNL*), xyloglucan endotransglucosylase/hydrolase (*XTH27*), protein NRT1/PTR family 4.3 (*NPF4.3*), beta-galactosidase-like (*BGAL*), pectin methlyesterase inhibitor (*PMEI*), lipid transfer protein (*EARLI-1*), GDSL esterase/lipase (*GDSL*) and aquaporin TIP1-1 (*TIP1-1*), involved in asparagine metabolism, nitrate or peptide transport, sugar metabolism, cell wall, fatty acid metabolism and transport, water transport and so on. After rehydration, the expression of these genes was higher or lower than that of the control and the previous drought stress, which may play an important role in the process of recovery after drought.

### 2.7. Drought-Hardened DEGs

Drought stress can produce trainable effect, showing stronger drought resistance to subsequent drought stress, and some genes will also be trained, showing a greater up- or downregulation than the initial stress under subsequent stress. There were 252 DEGs (136 up and 116 down) between the two treatments of RD1 and D1, which have similar drought intensity, and 134 DEGs (half up and half down) between similar drought intensity RD2 and D2. They were mainly involved in photosynthesis, signal transduction, sugar metabolism, protease and protease inhibitors, flavonoids and isoflavonoids metabolism, transporters, transcription factors, etc. (Table S4).

**Figure 6:**
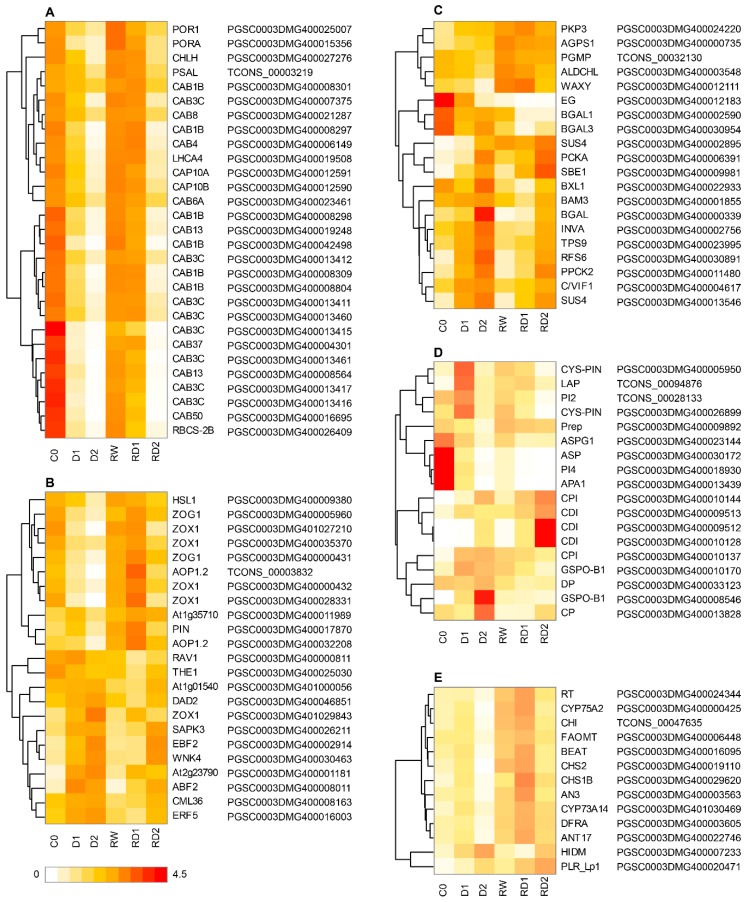
(**A**) The photosynthesis-related genes; (**B**) The hormone signal transduction related genes; (**C**) The glucose-metabolism-related genes; (**D**) The protease and protease inhibitor genes; and (**E**) The flavonoid-synthesis-related genes which were trained by drought.

After the first drought hardening, the expression levels of most photosynthesis-related genes in RD1 were higher than in D1 and also were slightly higher in RD2 than in D2 (Figure 6). This showed that the depressive level of photosynthesis was reduced under re-drought.

The hormone signal transduction related drought-hardened genes, which reached higher expression level in re-dehydration than that of the first drought, were mainly downregulated during the first drought stress and upregulated in the second drought. These genes are mainly related to zeatin synthesis, such as zeatin O-glucosyltransferase (*ZOG*), zeatin O-xylosyltransferase (*ZOX*), or gibberellin synthesis, such as 2-oxoglutarate-dependent dioxygenase (*AOP*) and auxin-signaling-related gene protein PIN-LIKES (*PIN*). Their expressions were affected in the initial drought and were induced during re-drought, which might enhance the drought resistance in the subsequent drought in potato. The genes with lower expression under second dehydration than the first were mainly upregulated under both drought stresses and mainly include kinase and transcription factors related to ethylene and ABA signal transduction, such as ABRE binding factor (*ABF*), serine/threonine-protein kinase SAPK3, ethylene-responsive transcription factor 5, AP2/ERF and B3 domain-containing transcription factor RAV1 (*RAV1*) and EIN3-binding F-box protein.

The protease inhibitors of the hardened genes were mainly upregulated under drought stress, while the protease genes were reversed. Flavonoid- and isoflavonoid-metabolism-related genes, such as flavonoid 3′,5′-methyltransferase-like, flavonoid 3′,5′-hydroxylase and dihydroflavonol-4-reductase-like, were upregulated in mild drought, downregulated in severe drought and higher in re-drought than in the first drought, indicating they could have a stronger response in subsequent drought after drought hardening.

The expression of hardened genes related to glucose metabolism is diverse. The responsive intensity of some genes in the second drought were lower than that in the first drought, such as sucrose synthase (*SUS4*), phosphoenolpyruvate carboxylase kinase 2-like (*PPCK2*), galactinol-sucrose galactosyltransferase 6 (*RFS6*), *BGAL*, etc. Also, the expression of some genes in the second drought was significantly higher than that in the control and the first drought, such as pyruvate kinase isozyme G (*PKP3*), glucose-1-phosphate adenylyltransferase large subunit 1 (*AGPS1*), phosphoglucomutase (*PGMP*), *ALDCHL* and *WAXY*, indicating the diversity of glucose-metabolism-related genes in the drought hardening.

### 2.8. Real-Time qRT-PCR Verification of DEGs

**Figure 7:**
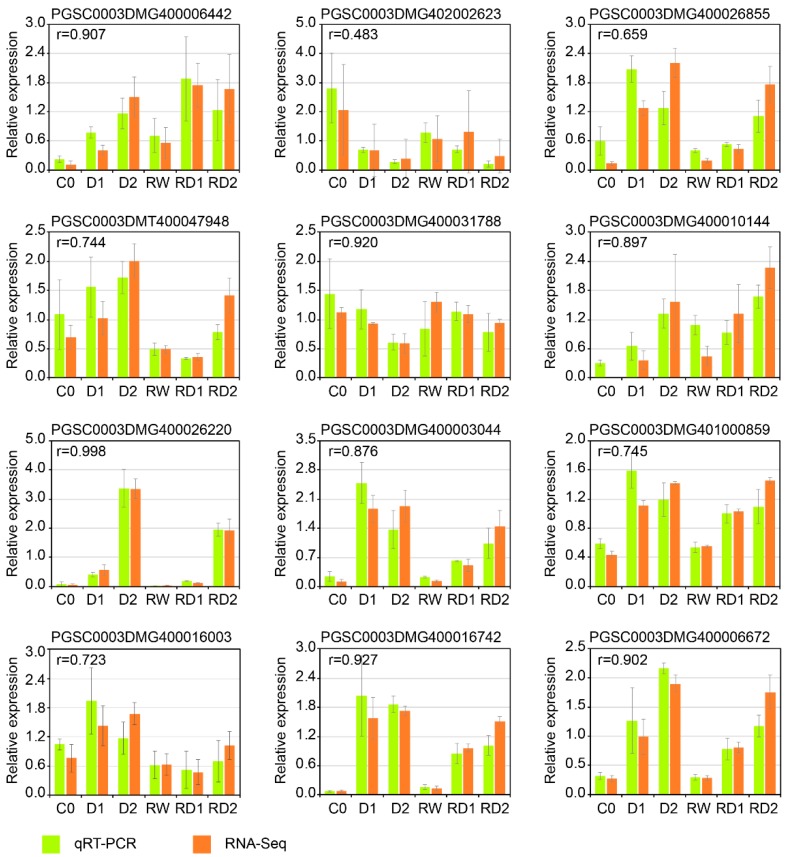
The expression analysis of 12 DEGs by qRT-PCR (yellow green) and RNA-seq (dark orange). The Pearson’s correlation coefficients (r) are labeled at the upper left corner. All the expression values of each gene were normalized by dividing the expression means of six treatments.

In order to verify the validity of transcriptome sequencing results, 12 DEGs were randomly selected for qRT-PCR validation. The expression trends between qRT-PCR and RNA-seq of each selected gene were similar, indicating that the transcriptome data are highly reliable (Figure 7).

## 3. Discussion

### 3.1. Perception and Transmission of Drought Stress Signals

**Figure 8:**
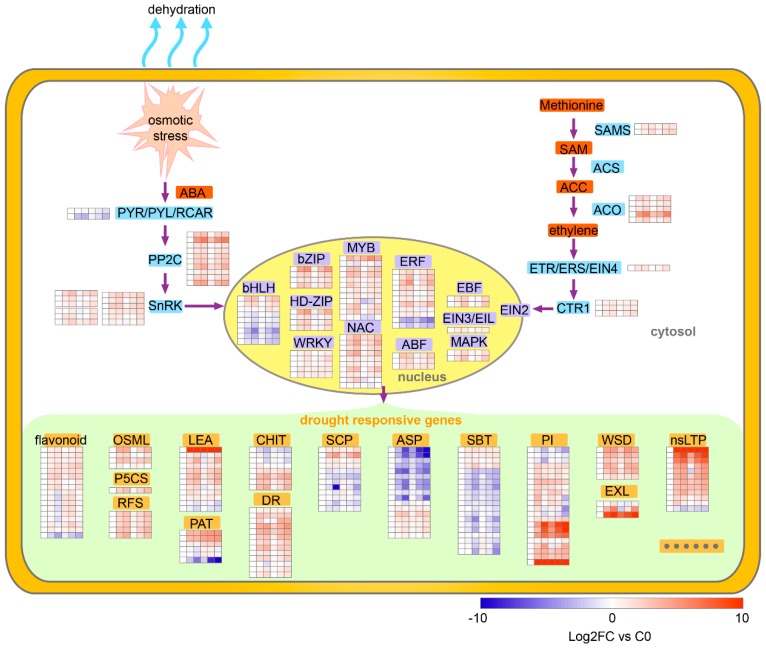
The schematic representation of the main process of drought response in potato.

The regulation of plant drought tolerance includes not only the key signaling metabolites and hormones, but also their activity-regulating proteins, such as kinases/phosphatases and TFs [13]. ABA is an important signal molecule in response to drought stress. Under drought stress, ABA signal regulates ABA signal pathway by inhibiting the phosphatase activity of PP2Cs protein through its receptor PYL protein family [20,21]. *PP2C* and *SnRKs* were upregulated after drought stress in potato. When exposed to drought again, although the expressions of most of them were slightly lower than the initial drought-induced expression, they were still upregulated by re-drought (Figure 8). They might be involved in the early response of plants to drought and affected by the drought hardening, which was similar to the ABA signal in *Arabidopsis* and rice participating in drought memory response after repeated drought treatments [13,22]. It is suggested that ABA signal plays an important role in the response of drought resistance and also in the improvement of subsequent drought resistance in plant. Ethylene synthesis could be induced by drought, and ethylene regulates the response to drought by activating the transcription factor of ethylene response factors (ERFs) [23,24]. In potato, the kinase and transcription factor related to ethylene signal were induced by drought, similar to ABA signal, but jasmonate (JA) signal was more obvious in rice [13], suggesting the difference of drought response and drought hardening mechanism between the two species. Strangely, auxin and zeatin signal related genes were inhibited in the first drought stress but induced in the second drought (Figure 6), indicating that they might be more effective for drought resistance in the second drought.

Transcription factors are essential in many signal transduction pathways in plants [25]. Many transcription factor families, such as *MYB*, *bZIP*, *bHLH*, *WRKY*, *NAC*, *ERF* and *DREB*, are involved in plant drought-resistance as key regulators in signal transduction pathway [9,26]. There were 178 differential expression transcription factors in JSY potato after drought stress, accounting for 4.7% of the drought-response genes (3754) and mainly involving 11 gene families, including *bHLH*, *bZIP*, *HD*-*ZIP*, *WRKY*, *MYB*, *NAC*, *ERF*, *ABF*, *EBF*, *EIN3/EIL* and *MAPK* (Figure 8). Most of them were upregulated, except for the majority of *bHLH*, which were downregulated after drought treatment, and some of them were upregulated or downregulated under re-drought more than those under the first stress, showing a drought memory effect [13,14]. It implies that they have important functions in drought response and hardened drought-resistance in potato. Recent studies have revealed that *ERF*, as a downstream component of ethylene signaling, is a key regulatory hub under abiotic stress in a variety of plants, integrating ethylene, ABA, JA and redox signaling, in response to drought, salt, light stress, cold and heat treatment [27]. Similar to the result, 9 of 11 *ERFs* were significantly upregulated after drought stress, highlighting their positive regulation of potato drought in this study.

Furthermore, transcription factors regulated the expression of drought-resistance genes in the present study. For example, flavonoid synthesis and metabolism genes, osmotic-adjustment-related genes (e.g., *P5CS*, *RFS* and *OSML*), protease inhibitors (*PI*), disease-resistance-related genes (*DR*), late embryogenesis abundant (*LEA*) proteins and wax synthesis and transport-related genes (*WSD*, *nsLTP*) were mostly upregulated, while the most of proteases (*ASP*, *SBT*) and peptidase (*SCP*) were downregulated. The upregulation of osmotic-adjustment-related genes could correspond to the osmotica, such as trehalose, raffinose, sucrose and melibiose, increasing after drought treatment in potato, as reported by Mane et al. [28]. Drought could also enhance the expression levels of flavonoid biosynthetic genes and increase total anthocyanin contents in a potato cultivar Huata Colorada [29]. A former metabolite profiling analysis showed the contents of proline and raffinose were increase in all the four experimental cultivars under drought stress [30]. These researches also implied that drought could regulate the osmotic adjustment substances and flavonoids by regulating the transcription of related genes.

Thus, drought induced the synthesis of ABA, ethylene and other endogenous hormones in potato and then affected the expression of genes related to flavonoids, disease resistance, LEA and wax in the downstream.

### 3.2. Wax Synthesis and Transport Involved Drought Resistance in Potato

Plants can cope with drought stress in a variety of ways, among which, increasing the accumulation of wax plays an important role in drought resistance. After synthesis in endoplasmic reticulum (ER), the wax was transported to the epidermis through the combination of wax transport proteins (LTP) and hydrophilic cell wall [31]. Differently from previous results, under drought stress, O-acyltransferase *WSD1*-like genes involved in wax synthesis showed a significant upregulation. The vast majority of nonspecific lipid-transfer proteins (*nsLTP*) were also intensely upregulated in JSY (Figure 8). The *nsLTP* may be involved in wax and/or cutin monomer transport [11,32,33]. The expression of a *nsLTP* in a drought-resistant potato material, Tajfun, was higher than drought-sensitive varieties and it could improve drought resistance in *Arabidopsis* [11]. The upregulation of *nsLPT* and *WSD1*-like genes under drought stress indicated that the waxy synthesis of JSY was active under drought stress. The accumulation of wax on leaf surface can reduce nonstomatal water loss [34,35,36]. The accumulation of wax in JSY may be an important mechanism of its resistance to drought.

### 3.3. Photosynthesis Was Hardened by Drought in Potato

Green plants use the chlorophyll binding protein (*LHC*) bound to PSI and PSII on the thylakoid membrane to accept solar energy and ultimately assimilate CO_2_. Chlorophyll a–b binding protein (*LHCB/CAB*) belongs to PSII and its expression is mainly regulated by environmental and developmental factors, mainly including light [37], circadian rhythm [38], abscisic acid [39] and other factors. Plant regulation of *LHCB* protein expression is considered to be one of the important mechanisms for plants to regulate chloroplast function in response to environmental challenges [38]. Under drought stress, plenty of *LHCBs* (*CAB*), protochlorophyllide reductase (the key enzyme of plant chlorophyll synthesis) and other genes involved in photosynthesis were significantly downregulated in JSY leaves, indicating that they participated in the early response of potato to drought (Figure 6). Photosynthesis, together with cell growth is the main process affected by drought [40]. The decrease in the expression level of the photosynthesis-related genes of JSY under drought stress indicates that plant water deficiency inhibits photosynthesis, and the degree of decline is greater with the increase of plant dehydration. However, the expression level of photosynthetic-related genes in the second drought was significantly higher than that in the first drought. The results showed that the inhibition of plants under the second drought was less than that under the first drought stress. This may be due to drought hardening working to improve the drought resistance of plants to a certain extent [12,13]. Early drought treatment had a trained effect in drought-resistance improvement in potato, which could show a certain drought resistance when encountering drought subsequently after rehydration.

### 3.4. Drought Hardening

Plants under drought stress can adapt to drought by physiological response adaptation and drought-adaptive structure, [38]. For example, the plants have greater leaf surface wax under arid conditions than under irrigated conditions [41]. A pretreatment of drought acclimation cycles followed by drought stress could induce thicker cuticular layer [42]. Under drought stress, plants adapt to drought by regulating soluble sugars, free amino acids, proline and ions in cells for osmotic adjustment [43]. Most of expansin-like B in potato, which has the potential of multistress resistance, can be upregulated under a variety of stresses, including drought [44]. In this study, wax synthesis and transport genes were active in drought. *P5CS*, the key enzyme of proline synthesis related to proline enrichment, was upregulated by 3.3 times (log2FC) under drought stress. LEA protein genes involved in osmotic adjustment were induced by drought in quantity, especially *DHN* and *ASR* subfamilies [45]. Expansin can loosen cell walls and reduce membrane damage during dehydration [46]; expansin-like B was also upregulated under drought stress (Table S2). These cellular structures and intracellular material changes may also play positive roles in subsequent drought stress.

Not only is drought hardening manifested in the structure and inclusion components, but also a lot of genes will be trained by previous drought stress; when drought occurs again, they will show higher or lower expression than the initial drought. For example, photosynthesis-related *CAB*, zeatin-synthesis-related *ZOX*, *ZOG*, gibberellin-synthesis-related *AOP*, protease inhibitor *CDI*, flavonoid synthesis- and metabolism-related genes were significantly higher in the second drought stress than in the first drought (Figure 6). This phenomenon is also considered to be drought memory [13,14]. Studies have shown that plant memory effects on stress are related to Ser5P polymerase II and histone modifications [14]. Drought hardening can make plants adapt to the subsequent drought and regulate the response of genes to suffer repeated drought, which has a positive effect on crops cultivated in the field to resist natural irregular drought. The mechanism which leads to drought memory in plant requires further research.

## 4. Materials and Methods

### 4.1. Plant Material

**Figure 9:**
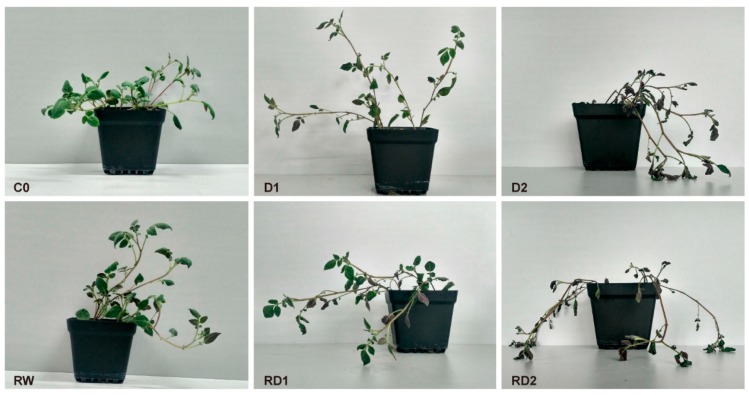
Phenotypes of Jancko Sisu Yari (JSY) under drought stress, rehydration and re-dehydration. C0 is the control of normal watering; D1 is the first mild drought stress; D2 is the first severe drought stress; RW is one day after rehydration; RD1 is the mild re-drought stress; and RD2 is the severe re-drought stress.

Jancko Sisu Yari (JSY), a drought tolerant diploid potato of *Solanum ajanhuiri* Juz. & Bukasov (CIP 706205), was used as the experiment material. The rooted in vitro seedlings of JSY were transplanted into the mixture of perlite and nutrient soil following culture for 1 month. Then the seedlings were water controlled until the soil water content (SWC) decreased to 20% and wilting of leaves occurred; this condition was termed as the mild drought stress (D1). The day after D1 sampling, SWC was decreased to 10% and leaves completely curled and wilted, which was termed as severe drought stress (D2). Then, the drought-stricken seedlings were watered to SWC of 80%. After one day of recovery, the curled leaves were re-spread entirely. The leaves were sampled as re-watered leaves (RW). A re-dehydration treatment was performed for the recovered seedlings. When SWCs were decreased to 20% and 10% again, the samples were defined as RD1 and RD2. Seedlings without water deficiency were used as controls (C0) (Figure 9).

### 4.2. RNA-Seq

The leaves of C0, D1, D2, RW, RD1 and RD2 were collected, frozen in liquid nitrogen and stored at −80 °C. Three biological replicates were performed for each of the six sample groups. Total RNA was extracted using RNAiso Plus reagent (Takara, Beijing, China). RNA-seq of mRNA was performed using Illumina HiSeq^TM^ 4000 by Genedenovo Biotechnology, Co., Ltd. (Guangzhou, China).

### 4.3. Alignment of RNA-seq Reads onto Reference Genome and Expression Analysis

After sequencing, high-quality clean reads were obtained by removed reads containing adapters, the all A bases reads, the reads containing more than 10% of unknown nucleotides (N) and the reads containing greater than 50% of low-quality (Q-value ≤ 20) bases. The alignment software TopHat2 (v2.1.1) [47] was used to map the reads to the reference genome *S. tuberosum* Group Phureja DM1-3 PGSC v4.03 [48]. Gene expression level analysis was based on FPKM (fragments per kilobase per million reads) value. Differential expression of genes between each two treatments was analyzed using edgeR package [49]. Differential expression genes (DEGs) were defined with the threshold |log2FC| ≥ 1 and FDR ≤0.05.

### 4.4. GO and KEGG Enrichment

Gene ontology (GO) functional classification enrichment was performed via WEGO (http://wego.genomics.org.cn/). GO categorizations were divided into hierarchies of molecular function, biological process and cellular component. KEGG pathway (https://www.kegg.jp/kegg/pathway.html) enrichment of DEGs were performed by comparing to the entire reference gene. The *p*-values of both GO and KEGG enrichment analyses were adjusted by Bonferroni correction, and the threshold of significance was *p* ≤ 0.05.

### 4.5. qRT-PCR

The real-time quantitative PCR (qRT-PCR) program was used for verifying the expression levels of RNA-seq. Total RNAs were reverse-transcribed using PrimeScript™ RT reagent Kit with gDNA Eraser (Takara, Beijing, China). qRT-PCR was performed in 20 μL reaction mixture with TB Green^®^ Premix Ex Taq™ II (Takara, Beijing, China) on Roche LightCycler 96 (Roche Diagnostics, Basel, Switzerland). cDNA of C0, D1, D2, RW, RD1 and RD2 were used as templates. All the primer sequences used in this study are listed in Table S5. The qRT-PCR program was set up for 30 s preincubation at 95 °C, 2-step amplification of 45 cycles at 95 °C for 5 s and 60 °C for 5 s, following a 60 to 97 °C melting curve analysis at the final step. Three independent biological repetitions and three parallel reactions were conducted in qRT-PCR. Potato elongation factor-1alpha (*EF1α*) was used as the reference gene [50].

## Figures and Tables

**Table 1:** The summary of differentially expressed genes (DEGs).

Comparison	DEG	Upregulation	Downregulation
C0–D1	1329	726	603
C0–D2	3203	1857	1346
D2–RW	2096	1053	1043
RW–RD1	461	239	222
RW–RD2	1772	935	837
C0–RW	308	138	170

## Supplementary Materials

Supplementary materials can be found at https://www.mdpi.com/1422-0067/21/1/159/s1. Table S1. The overview of transcriptome sequencing results. Table S2. Drought responsive gene in potato (C0 vs. D1). Table S3. DEGs after rehydration. Table S4. Drought hardened DEGs. Table S5. Primers for qRT-PCR analysis.
