# Supplementary material for: Transcriptome Response to Drought, Rehydration and Re-Dehydration in Potato
Source: Int J Mol Sci. 2019 Dec 25;21(1):159. doi: 10.3390/ijms21010159 (PMC6981527; doi:10.3390/ijms21010159)
Supplement: Supplementary file 1 [file ijms-21-00159-s001.zip › Table S5.docx]

**Table S5. Primers for qRT-PCR analysis.**

| **Gene ID** | **Forward primer (5'→3')** | **Reverse primer (5'→3')** | **Symbol** |
| --- | --- | --- | --- |
| PGSC0003DMG401000859 | GCCTTCAGCTTTCCACCATCAAAC | GAGCAAAGCTTGGAAATCCCTCTTC | CPY |
| PGSC0003DMG400003044 | CAACCAATCCTAGTGGAGGGAAATG | GCTATAGGCATCAGGGCATCTTTG | TPM-1 |
| PGSC0003DMG400006442 | GAGCTAGCCAACGGTGCAGATTTA | CCCAACATAGCAAATCTGCCATTCC | ELIP1 |
| PGSC0003DMG400006672 | CTAGTGGCTCTCCTGTCGAGATATG | GAGCACAATGGCTTCCTGAGTAG | SUS2 |
| PGSC0003DMG400016003 | GACAGAGCAGCATTTAGGCTCAG | CACCACAACTCTGTCATCATTCTCC | ERF5 |
| PGSC0003DMG400016742 | CCGAGTACGGAAGCTGCAAGAAG | GCAGCGATAGTGGCAGGGAAACAA | PP2C |
| PGSC0003DMG400026855 | CCCTGGATTCGGTGTCATCACAAAC | CACCAGGACTAACTCCAAGAATTCC | CHN50 |
| PGSC0003DMG400010144 | CCTCTCTTTGGTTGCCTTTGCTC | CAAGTATACGGCTCCGGCCCC | CPI |
| PGSC0003DMT400047948 | GTGCTGGATGTGGTGCTTGTTATCAGA | CTTGCCTTTGTAATCACAAGGAACTCTT | EXLA1 |
| PGSC0003DMG400026220 | CCCTGATTGCTATGGAACCCCTAGTG | TGGTCACCTTCACCATGATCTGTTAC | EXLB1 |
| PGSC0003DMG402002623 | GCTGAGATTACTGTCAGGAGTATGG | GCTGACCACTAGCAACAACGATGTC | LEA2-40 |
| PGSC0003DMG400031788 | CCGTTTCTGCTTTACTTAGCAGGCG | GAGCTCAGCAGCGTCAATCTCATTTG | LEA3-3 |
| StEF1α | CTGCACTGTGATTGATGCCCCTGGT | CTTCGGGGTGGTAGCATCCATCTTGT | EF1α |
